# Effects of Endohedral Gd-Containing Fullerenols with a Different Number of Oxygen Substituents on Bacterial Bioluminescence

**DOI:** 10.3390/ijms25020708

**Published:** 2024-01-05

**Authors:** Evsei A. Stepin, Ekaterina S. Sushko, Natalia G. Vnukova, Grigoriy N. Churilov, Anastasia V. Rogova, Felix N. Tomilin, Nadezhda S. Kudryasheva

**Affiliations:** 1 Biophysics Department, School of Fundamental Biology and Biotechnology, Siberian Federal University, 660041 Krasnoyarsk, Russia; stepin-kirill@mail.ru (E.A.S.); kkovel@yandex.ru (E.S.S.); 2 Institute of Biophysics SB RAS, FRC KSC SB RAS, 660036 Krasnoyarsk, Russia; 3 Institute of Physics SB RAS, FRC KSC SB RAS, 660036 Krasnoyarsk, Russia; nata_hd@rambler.ru (N.G.V.); churilov@iph.krasn.ru (G.N.C.); felixnt@gmail.com (F.N.T.); 4 Department of Solid State Physics and Nanotechnology, School of Engineering Physics and Radioelectronics, Siberian Federal University, 660074 Krasnoyarsk, Russia; 5 Department of Physical and Inorganic Chemistry, School of Non-Ferrous Metals and Materials Science, Siberian Federal University, 660025 Krasnoyarsk, Russia; arogova1927@gmail.com; 6 Laboratory for Digital Controlled Drugs and Theranostics, FRC KSC SB RAS, 660036 Krasnoyarsk, Russia

**Keywords:** endohedral fullerenol, gadolinium, bioluminescence, bacterial bioassay, toxicity, enzymatic bioassay, reactive oxygen species, density functional tight binding method, fourier-transform infrared spectroscopy

## Abstract

Gadolinium (Gd)-containing fullerenols are perspective agents for magnetic resonance imaging and cancer research. They combine the unique paramagnetic properties of Gd with solubility in water, low toxicity and antiradical activity of fullerenols. We compared the bioeffects of two Gd-containing fullerenols with a different number of oxygen groups—20 and 42: Gd@C_82_O_20_H_14_ and Gd@C_82_O_42_H_32_. The bioluminescent bacteria-based assay was applied to monitor the toxicity of fullerenols, bioluminescence was applied as a signal physiological parameter, and bacterial enzyme-based assay was used to evaluate the fullerenol effects on enzymatic intracellular processes. Chemiluminescence luminol assay was applied to monitor the content of reactive oxygen species (ROS) in bacterial and enzymatic media. It was shown that Gd@C_82_O_42_H_32_ and Gd@C_82_O_20_H_14_ inhibited bacterial bioluminescence at >10^−1^ and >10^−2^ gL^−1^, respectively, revealing a lower toxicity of Gd@C_82_O_42_H_32_. Low-concentration (10^−3^–10^−1^ gL^−1^) bacterial bioluminescence activation by Gd@C_82_O_42_H_32_ was observed, while this activation was not found under exposure to Gd@C_82_O_20_H_14_. Additional carboxyl groups in the structure of Gd@C_82_O_42_H_32_ were determined by infrared spectroscopy and confirmed by quantum chemical calculations. The groups were supposed to endow Gd@C_82_O_42_H_32_ with higher penetration ability through the cellular membrane, activation ability, lower toxicity, balancing of the ROS content in the bacterial suspensions, and lower aggregation in aqueous media.

## 1. Introduction

Fullerene is an allotropic form of carbon, a spherically closed nano-sized structure that consists of triple-coordinated carbon atoms. High resistance to cycle breaking keeps its structure from decaying in various organic environments [1,2,3]. A lot of studies related to the medical application of fullerenes and their derivatives have been conducted [1,2,4,5,6,7]. Magnetic resonance imaging (MRI) and photodynamic therapy are the new and perspective fields for fullerene applications [8]. The possibility to modify the fullerene surface with numerous substitutes and to form nanocomposites of different structure provides a variation of their properties is a highly useful feature for target therapy [9,10,11].

Low solubility in water restricts the biological and medical applications of pristine fullerenes. However, fullerenes are capable of forming water-soluble derivatives, polyhydroxylated fullerenes, which are known as fullerenols. Hydroxyl groups affect the conjugation of π electronic system of fullerenols, changing their chemical and biological activity. The amphiphilic structure of fullerenol nanoparticles determines their affinity to lipid structures of membranes and water-solubility, due to their carbon cage and hydroxyl groups, respectively. Such structural properties account for the ability to act as catalysts in biochemical reactions. In addition, similarly to fullerenes, fullerenols are characterized by anti-radical properties [12,13,14,15]. Both these features define fullerenols as highly perspective drugs for anti-tumor treatment [5,16,17,18]. Due to the application potential, relations between the structural characteristics and the parameters of the biological activity of fullerenols are of special interest at present.

The biological activity of fullerenols with a different number of hydroxyl groups have been studied over the last decades [19,20,21,22,23]. The toxic and antioxidant effects of fullerenols were under consideration. However, different biological test objects did not provide any comparability of the fullerenol biological activity. Comparable conditions were provided by Eropkin et al. [24] and Kovel et al. [25,26]. The biological activity of a series of fullerenols, C_60_(OH)_12–14_, C_60_(OH)_18–24_, and C_60_(OH)_30–38_ was studied by Eropkin [24]. It was found that C_60_(OH)_12–14_ was insoluble in water and did not show any biological activity, while C_60_(OH)_18–24_ was soluble and showed maximum antiviral and protective properties. Fullerenols C_60,70_ with a similar carbon cage, but a different number of oxygen groups (10–12, 24–28, and 40–42) were under investigation in studies by Kovel et al. [25,26] using bioluminescence assays; lower toxicity and higher antioxidant activity were determined in the solutions of fullerenols with 24–28 oxygen substituents. It was suggested that the optimal ratio of polar and non-polar fragments in the fullerenol macromolecules is responsible for this effect. This ratio can be attributed to the numbers of carbon and oxygen atoms (C:O) in the fullerenol structure; in the optimal case this ratio should be close to 0.5.

The biological effects of fullerenols offer a vast field of study with a variety of approaches [5,27,28,29]. Therapeutic effects of fullerenols [5,14,30] are usually explained by their radical scavenging properties.

Recently, Gd-endoderivatives of fullerenols have grasped attention due to their prospects in MRI [31]. The majority of currently applied Gd-containing compounds contrast MRI agents, such as gadodiamide, gadopentetate dimeglumine, etc., are of restricted application in medicine due to their toxicity [32,33,34]. A potential solution to this problem may be associated with securing gadolinium inside the fullerenol carcass, which prevents the release of Gd^3+^ ions into the biological environment [35]. Gd-endoderivatives of fullerenols combine the paramagnetic properties of the gadolinium atom with the antiradical and catalytic ability of fullerenols. Hence, the fullerenolic properties of Gd-endoderivatives are of particular interest.

As fullerene derivatives act as antiradical agents, their effects on reactive oxygen species (ROS) in biological systems are of special significance. ROS is a group of chemical compounds that contain oxygen with one or more unpaired electrons on the outer orbital. ROS are known to be involved in a number of physiological reactions that occur in the organism and, thereby, are normally present in physiological concentrations in organisms in order to perform metabolic reactions [36,37]. ROS can act as pleiotropic physiological signaling agents [38] or an apoptosis factor [39,40,41,42]. In addition, ROS can enhance the cytotoxic effect of drugs on tumor cells [43,44]. Formation of ROS occurs both in pathological and physiological conditions. ROS are formed in several biological systems, such as the mitochondrial respiratory chain and electron transport chain of microsomes, during the transition of oxyhemoglobin to methemoglobin, in the processes of arachidonic acid metabolism, in the hypoxanthine-xanthine oxidase reaction, during the biosynthesis and oxidation of catecholamines, etc., [45]. A lot of processes associated with the oxidation of biological molecules are accompanied by the generation of ROS [45,46]. At concentrations above the physiological ones, ROS are highly toxic for biological systems, as they cause the degradation of structural proteins, lipids of cell membranes, and nucleic acids [47].

Hence, the physiological role of ROS in cellular processes is important; they are necessary for the normal functioning of biological systems, being produced and utilized in normal metabolic processes. Maintaining the ROS balance is a vital function of organisms. However, the excess of ROS might be indicative of metabolic destructions; ROS can suppress physiological functions under the conditions of extra production.

The application of bioluminescence-based assays is a convenient way to test biological activity of various compounds. The luminescence feature of the bioassays provides a proper registration of biological responses. The intensity of luminescence is a physiological assay parameter monitored in the course of the bioassay procedure. Luminous marine bacteria are applied for several decades [48,49,50,51,52,53] as a conventional bioassay system. High rates of analysis (1–3 min), ease of use, high sensitivity, and availability of instruments and reagents are the advantages of the luminescence bacteria-based assay. Since the luminescence registration is not time consuming, it can provide a proper number of experimental results under comparable conditions and, therefore, proper statistical processing. This advantage is highly important for biological assays since they are usually characterized by lower reproducibility than the chemical or physical ones. In addition, this advantage is important for low-intensity exposures, which can be usually described in terms of “stochastic effects”. In particular, the rapid response registration is useful for non-genetic mechanisms of low-intensity exposures [54].

Similar to other bioassays, main features of the bioluminescence assays are an integral response and non-additivity. These features imply that all effects of exogenous compounds are integrated in a change of physiological functions (here, luminescence intensity), and the effect of a sum of compounds in complex solutions can be not equal to the sum of individual effects of these compounds.

Applying the bioluminescence enzymatic assay is a relatively new trend in the toxicology research [55,56,57]. The assay is based on two coupled enzymatic reactions of luminous bacteria (presented in Section 3.2). There exists an approach to study a mechanism of toxic effects using a combination of bioluminescence cellular and enzymatic assay systems: the differences in the bioluminescence responses of these systems allow evaluating the contributions of the biochemical effects into the cellular responses, and can largely be associated with cellular membrane functions.

Previously, we demonstrated a high potential bacteria-based and enzyme-based bioluminescence assays as appropriate tools for studying and comparing the bioeffects of nanocompounds of different structures [25,26,54,57,58,59,60,61,62,63]. Humic substances (products of natural decomposition of organic matter in soils and bottom sediments) were the first natural bioactive macromolecules studied using this approach [58]. Later, the effects of gold nanoparticles were analyzed [59], the toxicity and antioxidant activity of a series of different fullerenol nanoparticles were evaluated and compared [25,26,60], as well as prooxidant properties of magnetite nanoparticles were demonstrated [62,63]. The role of ROS in the effects of several nanostructures [25,26,57,63] and radionuclides [64,65,66,67] on luminous bacteria and their enzyme reactions was studied. The aim of this paper is to compare the effects of two gadolinium-containing fullerenols with different numbers of oxygen-containing substituents—20 and 42 (Gd@C_82_O_20_H_14_ and Gd@C_82_O_42_H_32_, respectively) on bacteria-based bioluminescence assay. According to “the rule of 0.5-ratio of carbon and oxygen atoms” (discussed before), the second fullerenol should be characterized by the lower toxicity and higher antioxidant activity. Thus, the toxic and activated effects of fullerenols are under consideration in this study. Additionally, the effects of fullerenols on bioluminescence of the bacteria cells and their enzymatic reactions are compared, and the differences are attributed to cell membrane processes. The mediating role of ROS in the effects of fullerenols is considered, and quantum-chemical calculations are applied to verify the relationship between the bioeffects and the structural peculiarities of fullerenols. Here, special attention is paid to the number of carboxyl groups on the surface of the fullerenol carbon cage.

## 2. Results and Discussion

We compared the effects of Gd@C_82_O_20_H_14_ and Gd@C_82_O_42_H_32_ on the bacterial and enzymatic bioluminescent assay systems. The quantitative characteristics of the toxicity and role of ROS in the toxic effects were under consideration.

### 2.1. Effects of Gd@C_82_O_20_H_14_ and Gd@C_82_O_42_H_32_ on Bioluminescence and on the ROS Content in the Bacterial Suspensions

#### 2.1.1. Effects of Gd@C_82_O_20_H_14_ and Gd@C_82_O_42_H_32_ on Bacterial Bioluminescence

**Figure 1:**
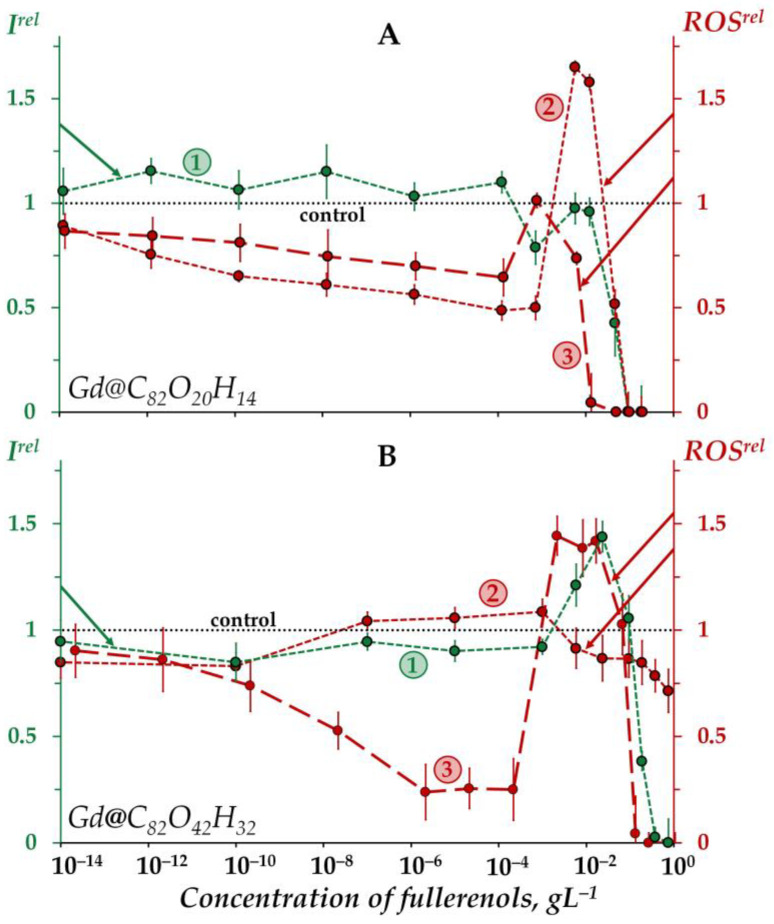
Relative bioluminescence intensity, *I^rel^* (1, green), relative ROS content in the bacterial suspension, *ROS^rel^* (2, red), and relative ROS content in distilled water, *ROS^rel^* (3, red) at different concentrations of fullerenols Gd@C_82_O_20_H_14_ (**A**) and Gd@C_82_O_42_H_32_ (**B**). The exposure time was 1 min. The concentration of ROS in the control bacterial suspension was 1.6 × 10^−6^ M and 4.5 × 10^−6^ M for Gd@C_82_O_20_H_14_ and Gd@C_82_O_42_H_32_, respectively; in distilled water—9 × 10^−7^ M and 3 × 10^−7^ M for Gd@C_82_O_20_H_14_ and Gd@C_82_O_42_H_32_, respectively. “Control” corresponds to the absence of fullerenols in the experimental solutions.

Figure 1 demonstrates the dependences of the bacterial bioluminescence intensity (curve 1), ROS content in the bacterial suspensions (curve 2), and ROS content in the bacteria-free media (curve 3) on the concentration of Gd@C_82_O_20_H_14_ (A) and Gd@C_82_O_42_H_32_ (B).

According to Figure 1A, the bioluminescence intensity of the bacteria (curve 1) does not show any evident response to Gd@C_82_O_20_H_14_ at low concentrations (<10^−2^ gL^−1^), the bioluminescence intensity being close to the control. At higher concentrations (>10^−2^ gL^−1^) a “harsh” drop in bacterial bioluminescence is observed, revealing the toxic effect of fullerenol. The effective concentration of fullerenol *EC*_50_ was determined to be 4 × 10^−2^ gL^−1^.

The “harsh” high-concentration (>10^−1^ gL^−1^) drop in the bacterial bioluminescence revealed the toxic effect of the other fullerenol, Gd@C_82_O_42_H_32_ (curve 1 in Figure 1B). The effective concentration of this fullerenol, *EC*_50_, was evaluated as 1.2 × 10^−1^ gL^−1^, which is close to that obtained earlier under similar conditions [57].

The *EC*_50_ value of Gd@C_82_O_42_H_32_ appears to be one order higher than that of Gd@C_82_O_20_H_14_. The comparison demonstrates the lower toxicity of Gd@C_82_O_42_H_32_, i.e., fullerenol with a higher number of oxygen substituents. This result confirms the “rule of 0.5-ratio of carbon and oxygen atoms” (discussed earlier in Introduction), which predicts a lower toxicity of fullerenol with the optimal ratio of polar and non-polar groups, i.e., Gd@C_82_O_42_H_32_. It should be noted that the toxic effect of this fullerenol appears to be the lowest one among all fullerenols studied earlier under similar conditions [25,26].

The bacterial luminescence response to Gd@C_82_O_42_H_32_ (Figure 1B, curve 1) corresponds to the conventional “hormesis” model [68,69,70,71]. It is known that this model includes, in the broadest case, three stages of the biological dose-dependent response: stress recognition (I), activation (II), and inhibition of organismal functions, i.e., toxic effect (III). As a concept, hormesis always involves favorable biological responses to low exposures of stressors [72,73]. According to Figure 1B, the dependence of the bacterial bioluminescence intensity *I^rel^* on the Gd@C_82_O_42_H_32_ concentration (curve 1) includes three stages mentioned above: (I) slight inhibition (*I^rel^* < 1) at <10^−3^ gL^−1^, (II) reliable activation (*I^rel^ > 1*) at 10^−3^–10^−1^ gL^−1^, and (III) inhibition (*I^rel^* < 1) at >10^−1^ gL^−1^. In contrast to Gd@C_82_O_42_H_32_ (Figure 1B, curve 1), the exposure of bacterial cells to Gd@C_82_O_20_H_14_ did not result in the bioluminescence activation (Figure 1A, curve 1).

The toxic effects of fullerenols are associated with complex multiple processes which result in the inhibition of membrane and intracellular processes by fullerenol nanoparticles, as previously discussed [25,26,57,60]. The explanation of the toxic effects should involve the possibility of aggregate formations, studied in detail previously in [31,74,75,76] using the example of endohedral fullerenol Gd@C_82_(OH)_22_; polyanion nano-aggregation into clusters in aqueous solutions was also demonstrated. This approach assumes that the high-concentration toxic effects (*I^rel^* < 1 at >10^−2^ gL^−1^ for Gd@C_82_O_20_H_14_ and >10^−1^ gL^−1^ for Gd@C_82_O_42_H_32_, Figure 1A,B, curves 1) are produced by fullerenol aggregates.

The difference in the toxic effects of Gd@C_82_O_42_H_32_ and Gd@C_82_O_20_H_14_ should be attributed to the different number of oxygen groups, which provide a different ratio of polar and non-polar fragments on the surface of the fullerenol carbon cage. The presence of carboxyl groups in the structure of Gd@C_82_O_42_H_32_ (in contrast to the second fullerenol) is determined by fourier-transform infrared (FTIR) spectroscopy and confirmed theoretically in our study (see Section 2.3); their polar characteristics are known to be maximum among all possible oxygen substituent types. The structural difference regulates the aggregate formation. (The dependence of the aggregation ability of the Gd-containing fullerenol Gd@C_82_(OH)_22_ on the polarity of its surface was studied previously [74,75,76]). We suspect that extra oxygen groups (carboxyl, particularly) can account for the unequal membrane permeability due to the formation of conductive defects. Previous studies [77,78] form the basis for this explanation via the involvement of aggregation into the cell entering mechanisms.

Additionally, the higher penetrative ability of Gd@C_82_O_42_H_32_ through the cell membrane may result in more effective stimulation of intracellular bioluminescence enzymatic processes. To confirm this suggestion, we studied the effects of two fullerenols on bioluminescence enzyme reactions as discussed below in Section 2.2.

Furthermore, we studied the toxicity of additional Gd compounds in a series of bioluminescence experiments; the conventional Gd-containing contrast agent gadodiamide and GdCl_3_ were chosen and the dependences of the bacteria bioluminescence intensity on the compound’ concentrations were analyzed at the initial time of exposure. We revealed a lower toxicity of the agents than that of the Gd-containing fullerenols under similar conditions. However, the toxic characteristics were found to change with the time of exposure, showing an increase in the toxicity. A similar toxicity time-course under exposure to europium (heavy atom, actinide, chemical analogue of gadolinium) was demonstrated previously in [79]. It should be noted that the toxic effects of heavy metals are a well-known phenomenon in toxicology, involving the bacteria bioluminescent bioassay [49,53,80]. The toxicity of heavy metals is connected with the low ionization energy of atomic outer electrons, and, hence, high catalytic activity of heavy metals. Thus, the results show the necessity of further comprehensive research to compare the time-courses of toxic characteristics of Gd-containing fullerenols and conventional MRI contrast agents under comparable conditions.

#### 2.1.2. Effects of Gd@C_82_O_20_H_14_ and Gd@C_82_O_42_H_32_ on the ROS Content in Bacterial Suspensions

Figure 1A,B (curves 3) demonstrate the dependences of the ROS content on the concentration of Gd@C_82_O_20_H_14_ and Gd@C_82_O_42_H_32_ in non-bacterial suspensions (water media). The dependences are of a non-monotonic character, involving two decay intervals in different concentration regions (<5 × 10^−4^ and >10^−1^ gL^−1^) and one maximum (10^−3^–5 × 10^−2^ gL^−1^). The decay intervals are the evidence of antiradical activity of the fullerenols, while the maxima can presumably be associated with the aggregation of nanoparticles, as mentioned above. The aggregation results in the reduction in the nanoparticle surface area which is responsible for the antiradical activity and, hence, ROS neutralization. According to the aggregation-based approach, the low-concentration ROS-decay (<5 × 10^−4^ gL^−1^) is provided by the single fullerenol macromolecules, while the high-concentration ROS-decay (>10^−1^ gL^−1^) is provided by the fullerenol aggregates.

The ROS peak in the concentration interval (5 × 10^−4^–5 × 10^−2^ gL^−1^) of Gd@C_82_O_42_H_32_ (Figure 1B, curve 3) can be attributed to the formation of “small aggregates” that can presumably be responsible for the peak of bacteria bioluminescence (Figure 1B, curve 1). Hence, we suppose that the coincidence of the fullerenol’ concentration ranges for activations of bacterial bioluminescence and ROS content (*I^rel^* > 1 and *ROS^rel^* > 1, curves 1 and 3 in Figure 1B) contributes to understanding the hormesis mechanism in the bacteria via physico-chemical processes of ROS formation in water media.

The low-concentration ROS decay and ROS maximum are more pronounced for Gd@C_82_O_42_H_32_ (Figure 1B, curve 3) as compared to Gd@C_82_O_20_H_14_ (Figure 1A, curve 3). This difference in the effects can be due to additional oxygen groups on the surface of Gd@C_82_O_42_H_32_, providing a higher ionic strength in water and affecting the antiradical and aggregation ability of fullerenols. Carboxyl groups, in the case of their presence on the surface of the fullerene carcass, are most effective among different oxygen-containing groups.

The concentration dependence of the ROS content for Gd@C_82_O_20_H_14_ in the bacteria suspension (Figure 1A, curve 2) corresponds to that in the bacteria-free media (Figure 1A, curve 3), i.e., it does not change dramatically upon the addition of bacteria to the fullerenol water solutions. The opposite effect was found for the other fullerenol, Gd@C_82_O_42_H_32_ (Figure 1B, curves 2,3): concentration-coursers of the curves 2 and 3 differed. Note that the concentration dependence of the ROS content for Gd@C_82_O_42_H_32_ in the bacteria suspension (Figure 1B, curve 2) correlated negatively with the one in the bacteria-free media (Figure 1B, curve 3); the correlation coefficient *r* was −0.47 in the range of 10^−8^–10^−1^ gL^−1^. We can conclude that the bacteria compensate for the lack of ROS in the outer water solution, thus keeping the ROS balance closer to the control in solutions of Gd@C_82_O_42_H_32_. Probably, this fullerenol stimulated the ROS production by the bacteria as a result of preferable penetration through the cell membranes and activation of intracellular processes.

The difference in the effects of Gd@C_82_O_20_H_14_ and Gd@C_82_O_42_H_32_ on the ROS content can be attributed to the structural peculiarity of Gd@C_82_O_42_H_32_, i.e., to the involvement of additional oxygen groups into the surface of the fullerenol macromolecule. The presence of the most effective polar groups (carboxyl) is proved theoretically in the current study (see Section 2.3).

### 2.2. Effects of Gd@C_82_O_20_H_14_ and Gd@C_82_O_42_H_32_ on Bioluminescence and on the ROS Content in an Enzymatic Solution

To evaluate the effects of fullerenols on intracellular enzyme processes we chose the bacterial bioluminescence enzymatic system. The bioluminescence intensity and the ROS content were monitored in the enzymatic solution at different concentrations of fullerenols.

#### 2.2.1. Effects of Gd@C_82_O_20_H_14_ and Gd@C_82_O_42_H_32_ on Bioluminescence in Enzymatic Solutions

**Figure 2:**
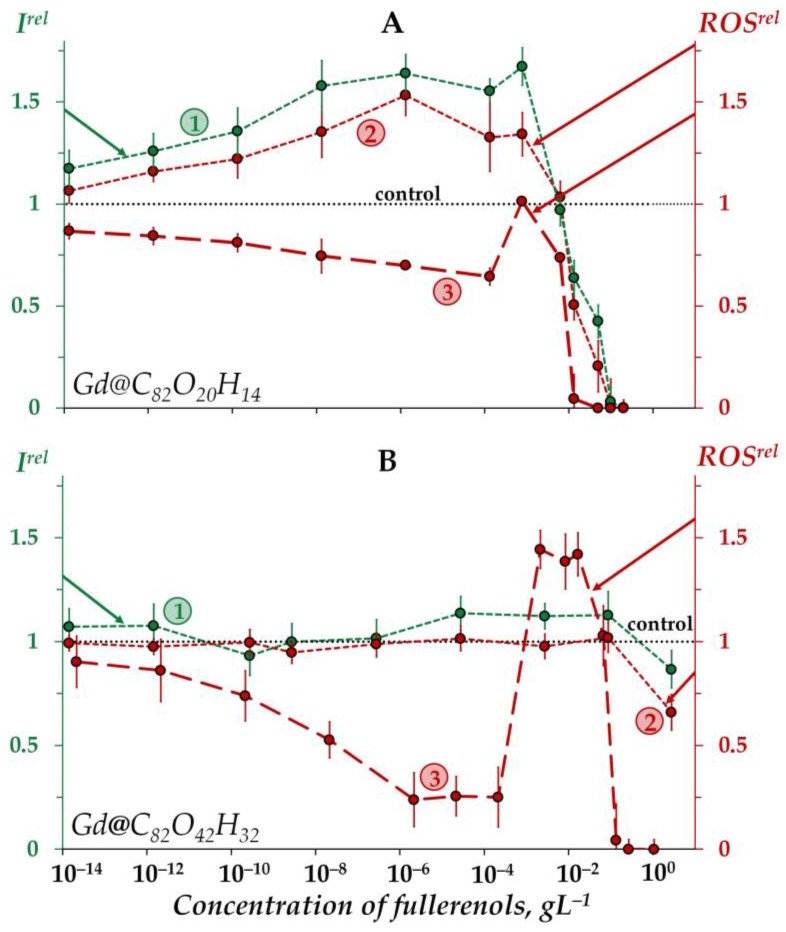
Relative bioluminescence intensity, *I^rel^* (1, green), relative ROS content in the enzymatic system, *ROS^re^*^l^ (2, red), and relative ROS content in distilled water, *ROS^rel^* (3, red) at different concentrations of fullerenols Gd@C_82_O_20_H_14_ (**A**) and Gd@C_82_O_42_H_32_ (**B**). The time of exposure was 1 min. The ROS concentration in the control enzymatic system was 2.5 × 10^−5^ M and 1.9 × 10^−5^ M for Gd@C_82_O_20_H_14_ and Gd@C_82_O_42_H_32_, respectively; in distilled water—9 × 10^−7^ M and 3 × 10^−7^ M for Gd@C_82_O_20_H_14_ and Gd@C_82_O_42_H_32_, respectively. The “control” corresponds to the absence of fullerenols in the experimental solutions.

Figure 2A shows an increase in the bioluminescence intensity (*I^rel^* > 1) of the enzyme system under exposure to Gd@C_82_O_20_H_14_ in the low-concentration interval <5 × 10^−3^ gL^−1^ (curve 1). Thus, the fullerenol demonstrates the catalytic activity in the bioluminescence system under the conditions applied. As discussed above, this fullerenol did not affect the bacterial bioluminescence intensity (Figure 1A, curve 1) in a similar concentration range. We can explain this difference by the non-penetration of this fullerenol into the cells.

Bioluminescence inhibition is evident at higher concentrations of the fullerenol (>5 × 10^−3^ gL^−1^).

The other fullerenol, Gd@C_82_O_42_H_32_, did not noticeably change (<20%) the bioluminescent intensity of the enzyme system, i.e., it did not reveal any valuable catalytic activity (Figure 2B, curve 1). Probably, a higher number of polar oxygen-containing groups in this fullerenol decreases its affinity to enzymes and, hence, its catalytic activity. We can conclude that the activation of bioluminescence by Gd@C_82_O_42_H_32_, (Figure 1B, curve 1) at concentrations of 5 × 10^−4^–10^−1^ gL^−1^ is not related to the activation of intracellular enzyme processes; the preference should be given to the mechanism of “small aggregate” formation, as discussed earlier in Section 2.1.2.

#### 2.2.2. Effects of Gd@C_82_O_20_H_14_ and Gd@C_82_O_42_H_32_ on the ROS Content in the Enzymatic Solution

As seen from Figure 2A, the concentration course of the bioluminescence intensity (curve 1) correlates with that of the ROS content (curve 2) in the enzyme system over the whole concentration range of Gd@C_82_O_20_H_14_. The correlation coefficient *r* turns out to be 0.98, evidencing that this fullerenol synchronically activates enzymatic processes in the system at lower concentrations (<5 × 10^−3^ gL^−1^), which results in the ROS formation. According to [81,82], the ROS group in the complex bioluminescence enzyme process involves peroxychemiacetal (intermediate II in the bioluminescence reaction of bacterial luciferase (R2, Section 3.2)) and hydrogen peroxide (product of oxidation of reduced flavin). The former corresponds to the light-emitting line of the process, while the latter corresponds to the dark lines.

At higher Gd@C_82_O_20_H_14_ concentrations (>5 × 10^−3^ gL^−1^), ROS decay is observed in the enzyme system (Figure 2A, curve 2), demonstrating the antiradical activity of the fullerenol. This neutralization of peroxide radicals by Gd@C_82_O_20_H_14_ is likely to be responsible for synchronic higher-concentration inhibition of the formation of bioluminescence peroxide intermediate (intermediate II in the bioluminescence reaction of bacterial luciferase) and, as a result, for the inhibition of bioluminescent reaction.

The other fullerenol, Gd@C_82_O_42_H_32_, did not reveal any changes in ROS content in the enzyme system under all the concentrations studied (Figure 2B, curve 2), the effect being similar to that for bioluminescence intensity (Figure 2B, curve 1).

An interesting point is to be noted if to compare the ROS content (*ROS^rel^*) in the enzymatic and enzyme-free media for both fullerenols (Figure 2A,B, curves 2 and curves 3, respectively). As is seen from the comparison of the curves, the addition of the enzymes to the water solutions of fullerenols increases the ROS content at small and moderate fullerenol concentrations (<10^−3^ gL^−1^), demonstrating the ability of biological enzymatic systems to balance the ROS content.

### 2.3. Theoretical Results

To understand and predict biological effects of fullerenols, we should know their structure involving key functional groups on the surface of their cages.

Fullerenols Gd@C_82_O_20_H_14_ and Gd@C_82_O_42_H_32_ were synthesized at different time of exposure to nitric acid (see Section 3.1), and, hence, we obtained fullerenols of irregular composition with different amounts of oxygen and hydrogen atoms on surface of the carbon cages. This makes it difficult to determine the atomic structure by X-ray electron diffraction, and thus, indirect methods such as FTIR and X-ray photoelectron spectroscopy (XPS) are widely used. These methods allow us to estimate the presence of functional groups and the approximate oxygen and hydrogen content on the fullerene cage. The characteristic bands in the experimental FTIR spectrum suggest that C=O, epoxy, -OH and -COOH groups are formed on the surface of fullerenols. In this case, the groups break the carbon cage. Different combinations of types and numbers of functional groups can lead to different mechanisms in biological systems. Therefore, models of C_82_O_x_H_y_ fullerenols where x = 20; y = 14 and x = 42; y = 32 should be constructed to explain different behavior. These models correspond to the fullerenols (Gd@C_82_O_20_H_14_ and Gd@C_82_O_42_H_32_, respectively), which were experimentally studied in Section 2.1 and Section 2.2.

#### 2.3.1. Theoretical Calculations of C_82_O_20_H_14_

**Figure 3:**
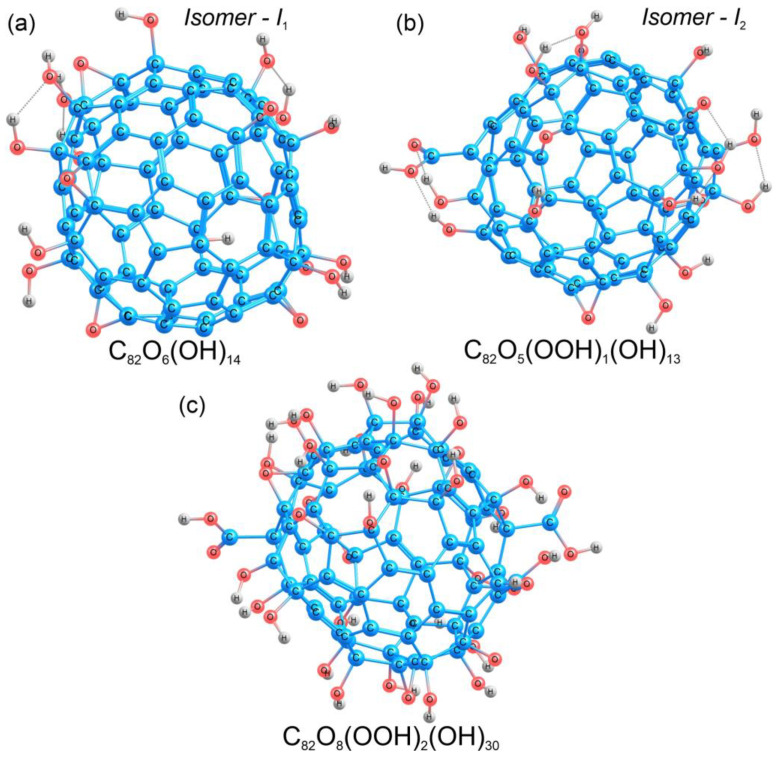
Atomic structures of the valence isomers of C_82_O_20_H_14_ (**a**,**b**) and the structure of C_82_O_42_H_32_ (**c**). Carbon, oxygen, and hydrogen atoms are shown in blue, red, and grey, respectively.

Fullerene Gd@C_82_ in the C_2v_ symmetry was taken as a starting point for the construction of different structural models (Gd@C_82_O_x_H_y_), since the experimental value of the FTIR spectra for this molecular geometry is known. To construct the model of Gd@C_82_O_20_H_14_, the ratio of hydroxyl (-OH) groups to oxygen was chosen to be ~1:3, in agreement with the experimental data (see Section 3.1), the total number of oxygen atoms was 20. The structure of metallofullerene Gd@C_82_ has a C_2v_ space group; thus, for the calculations, 20 oxygen atoms were distributed symmetrically on the carbon cage, and then, 14 hydrogen atoms were added to the oxygen atoms. Gadolinium was removed from the obtained models because, according to the experimental data, the spectra of gadolinium ions (700–800 cm^−1^) do not lie in the region of fullerenol functional groups. Next, two valence isomers of C_82_O_20_H_14_ were calculated, which were different in the composition and number of functional groups. The first isomer C_82_O_20_H_14_ (I_1_) contained 14 hydroxyl (-OH) and 6 carbonyl (C=O) groups (Figure 3a), distributed as eight pairs of carbonyls and hydroxyls, and two pairs of hydroxyls attached to adjacent carbon atoms. During the optimization of the geometry of the first isomer, all the carbonyl groups were converted to epoxide groups, maintaining the atomic structure of the carbon cage. As a result, the equilibrium structure of isomer I_1_ involved 14 hydroxyl (-OH) and 6 epoxide (C-O-C) groups (Figure 3a).

#### 2.3.2. Theoretical Calculations of C_82_O_42_H_32_

The atomic structure of Gd@C_82_O_42_H_32_, was modeled in the same way as in the case of C_82_O_20_H_14_ (Section 2.3.1). Forty-two oxygen atoms were distributed symmetrically over the entire surface of the carbon cage. Further, hydrogen was added to the oxygen atoms, resulting in different functional groups (hydroxyl, carbonyl, and carboxyl). For this compound, the band in the FTIR spectrum at around 1700 cm^−1^ indicated a carboxyl group [83], and thus, we built two carboxyl groups symmetrically on the opposite sides of the carbon cage. As a result, there were 30 hydroxyl (-OH), 8 epoxy (C-O-C), and 2 carboxyl (-COOH) functional groups in the C_82_O_42_H_32_ fullerenol model (Figure 3c). The detailed information on the atomic structure can be found in the Supplementary Materials (Cartesian coordinates, Figures S1–S3).

#### 2.3.3. Theoretical Infrared (IR) Spectra of C_82_O_42_H_32_

**Figure 4:**
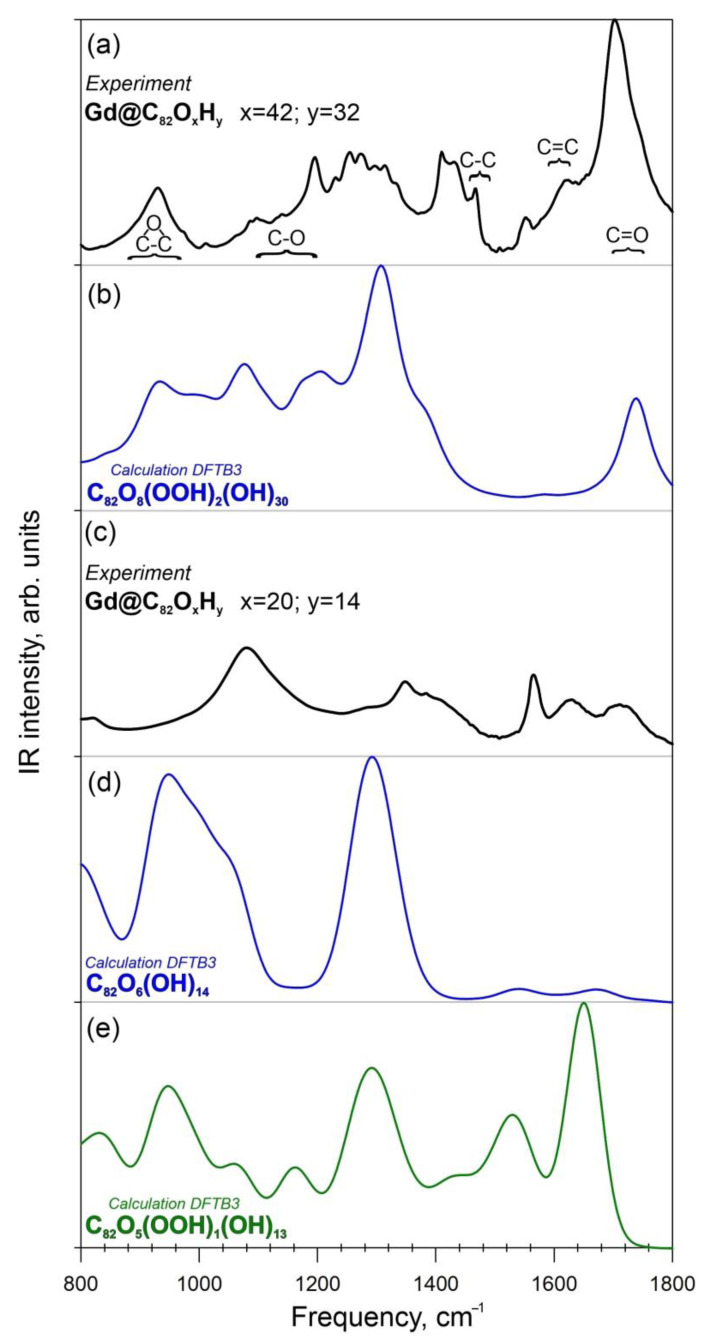
Experimental and theoretical IR spectra of Gd@C_82_O_x_H_y_. (**a**)—Experimental FTIR spectrum of Gd@C_82_O_42_H_32_; (**b**)—Theoretical IR spectrum of the compound C_82_O_42_H_32_; (**c**)—Experimental FTIR spectrum of Gd@C_82_O_20_H_14_; (**d**)—Theoretical IR spectrum of the I_1_-isomer C_82_O_20_H_14_; (**e**)—Theoretical IR spectrum of the I_2_-isomer C_82_O_20_H_14_.

The theoretical IR vibration spectra for the atomic structure of C_82_O_42_H_32_ calculated at the density functional tight binding method (DFTB3) level of theory are presented in Figure 4b. The comparison of the experimental FTIR spectrum (Figure 4a) with the theoretical data shows good agreement between the spectra in the region 850–1350 cm^−1^. The IR spectrum has characteristic bands in the regions of 1600–1670 cm^−1^ (carbon cage) and 1690–1720 cm^−1^ (-COOH groups) (Figure 4b). Thus, carboxyl groups were present in the compound (C_82_O_x_H_y_, x = 42 y = 32).

#### 2.3.4. Theoretical IR Spectra of C_82_O_20_H_14_

The comparison of the experimental FTIR (Figure 4c) spectra and theoretical IR spectra (Figure 4d) calculated at the DFTB3 level of theory shows good agreement between the spectral lines in the region 900–1300 cm^−1^, showing vibrational lines of epoxy, C-O, C-C and C=C groups (Figure 4d,e). Isomer I_1_, which contains only hydroxyl and epoxy functional groups, shows the best agreement between the calculated and experimental FTIR spectra (in the region 850–1350 cm^−1^) (Figure 4e). For isomer I_2_ (with carboxylic group), the similarity between the theoretical and experimental IR spectra can be seen. However, an intense band at 1700 cm^−1^ is present in the theoretical spectrum, in contrast to the experiment (Figure 4d). It can be concluded that the compound C_82_O_20_H_14_ lacks a carboxyl group.

#### 2.3.5. Theoretical Discussion

According to quantum chemical calculations of different models of fullerenols, we can conclude that several types of fullerenols can be formed during their synthesis. The main difference between the two fullerenols (Gd@C_82_O_20_H_14_ and Gd@C_82_O_42_H_32_) is the presence of carboxyl groups (Figure 3 and Figure 4). This should lead to different physicochemical properties of two fullerenols. Probably, the additional carboxyl groups increase electron affinity of Gd@C_82_ core. It is known that Gd-containing fullerenes are of ionic structure; the inner paramagnetic ion Gd^3+^ is encapsulated in the negatively charged carbon cage, thus forming a dipole charge-transfer complex Gd^3+^@C_82_^3–^ [35]. Now, ionic conjecture for Gd@C_82_ is accepted by the scientific community [84]. The additional carboxylic groups are able to affect the polarity of bioenvironment to a higher degree.

The calculations confirmed the involvement of carboxyl groups into the fullerenol structure, as determined experimentally by FTIR (Figure 4).

To sum up, the additional number of oxygen-containing groups on the surface of Gd@C_82_O_42_H_32_, involving carboxyl groups endows the fullerenol with particular chemical, biochemical and microbiological activity, as compared to Gd@C_82_O_20_H_14_. The structural peculiarities form the basis for speculations on the lower toxicity and activation ability of fullerenol Gd@C_82_O_42_H_32_, its higher penetrative ability through the cellular membrane, and aggregation in aqueous media.

## 3. Materials and Methods

### 3.1. Preparation of Fullerenols

A fullerene mixture was preliminary synthesized using carbon helium high-frequency arc plasma at 98 kPa [85]. The carbon soot contained about 4.8% of Gd@C_82_-fullerene. In order to enrich the extract of the fullerene mixture with endohedral metallofullerenes, the reaction of complexation with Lewis acids (TiCl_4_) was applied [86]. Fullerenoles were produced by boiling Gd@C_82_ in nitric acid (for 5 h and 8 h, respectively) which was followed by polynitrofullerenes hydrolysis at 85 °C [87,88,89]. The products were characterized by IR in the KBr matrix using a Fourier spectrometer VERTEX 70 (Bruker Optik GmbH, Ettlingen, Germany). The number of -OH groups was estimated by XPS using a UNI-SPECS spectrometer (SPECS Gmbh, Berlin, Germany) [88,89] (Supplementary Materials, Characterization of Gd-containing fullerenols, Table S1, Figure S5). Gd-endohedral fullerenols Gd@C_82_O_x_H_y_ where x = 20; y = 14 (Gd@C_82_O_20_H_14_) and x = 42; y = 32 (Gd@C_82_O_42_H_32_) were synthesized [90]. The assessment of water in the samples was performed by thermogravimetric analysis (synchronous thermal analysis device NETZSCH STA 449C Jupiter (NETZSCH-Geratebau GmbH, Selb, Germany) combined with a quadrupole mass spectrometer QMS 403C (NETZSCH-Geratebau GmbH, Selb, Germany). The amount of water was 1.63% in the sample of Gd@C_82_O_42_H_32,_ and 4.27% in the sample of Gd@C_82_O_20_H_14_. Results of thermogravimetric analysis are presented in the Supplementary Materials (Characterization of Gd-containing fullerenols, Figure S4).

### 3.2. Bioluminescence Assay Systems and Experimental Data Processing

The biological activity of Gd-containing fullerenols was evaluated using cellular and enzymatic bioluminescent assay systems: (1) bacterial assay, i.e., intact marine luminous bacteria *Photobacterium phosphoreum*, strain 1883 IBSO from the Collection of Luminous Bacteria CCIBSO 863, Institute of Biophysics SB RAS (Krasnoyarsk, Russia), and (2) enzymatic assay, i.e., enzyme preparation based on the system of coupled enzyme reactions catalyzed by NADH:FMN-oxidoreductase from *Vibrio fischeri* (0.15 a.u.) and luciferase from *Photobacterium leiognathi*, 0.5 mg/mL [91]. The enzyme preparation was produced at the Institute of Biophysics SB RAS (Krasnoyarsk, Russia).

The chemicals used were the following: flavinmononucleotide (FMN) and tetradecanal from SERVA, Heidelberg, Germany; nicotinamide adenine dinucleotide, disodium salt, reduced (NADH) from ICN Biochemicals, Costa-Mesa, CA, USA; sodium chloride (NaCl) from Khimreactiv, Nizhny Novgorod, Russia. The reagents were of chemical or analytical grade.

To prepare the enzymatic assay system we used 0.1 mg/mL of enzyme preparation, 4 × 10^−4^ M NADH, 5.4 × 10^−4^ M FMN, and 0.0025% tetradecanal solutions. The NADH, FMN and tetradecanal were dissolved in distilled water. The concentrations of NADH, FMN, and tetradecanal solutions in the experimental samples were 1.6 × 10^−4^ M, 5.4 × 10^−5^ M, 0.00025%, respectively.

The enzymatic assay system is based on the following coupled enzymatic reactions:

(R1) $$NADH+FMN\overset{NADH:FMN-oxidoreductase}{\to }FMN\cdot H^{-}+NAD^{+}$$

(R2) $$FMN\cdot H^{-}+RCHO+O_{2}\overset{luciferase}{\to }FMN+RCOO^{-}+H_{2}O+h\nu$$

For the cultivation of *P. phosphoreum* 1883 IBSO, the semisynthetic medium containing 10 gL^−1^ tryptone, 28.5 gL^−1^ NaCl, 4.5 gL^−1^ MgCl_2_∙6H_2_O, 0.5 gL^−1^ CaCl_2_, 0.5 gL^−1^ KCl, 3 gL^−1^ yeast extract, and 12.5 gL^−1^ agar was used. *P. phosphoreum* was plated on 25 mL of semisynthetic medium and incubated at 25 °C for 24 h (stationary growth phase corresponding to maximum bioluminescence) in an incubator (WIS-20R, WiseCube Laboratory Instruments, Wertheim, Germany). Prior to the experiments, bacteria were collected by pipetting 3% NaCl solution directly onto the agar to release bacteria. The 3% NaCl solutions were used to imitate a marine environment for the bacterial cells and to balance osmotic processes. The bacterial suspension was diluted to Abs_660_ = 0.025 and stored at 4 °C for 30 min to allow bioluminescence stabilization. The reagents for bacterial cultivation were: tryptone and yeast extract from Dia-M, Moscow, Russia; sodium chloride (NaCl) from Khimreactiv, Nizhny Novgorod, Russia; magnesium chloride hexahydrate (MgCl_2_ 6H_2_O), calcium chloride (CaCl_2_), and potassium chloride (KCl) from Pancreac AppliChem GmbH, Darmstadt, Germany; agar from Difco Laboratories, Detroit, MI, USA.

The biological effects of fullerenols on bioluminescence of the bacterial and enzymatic assay systems were characterized by the relative bioluminescence intensity, *I^rel^*:

*I^rel^* = *I_F_*/*I_contr_*, (1)

where, *I_contr_* and *I_F_* are the maximum bioluminescence intensities in the absence and presence of fullerenols, respectively.

Concentrations of fullerenols varied. The effective concentration of fullerenols inhibiting the bioluminescence intensity by 50% (*I^rel^* = 0.5), *EC*_50_, were determined to evaluate their toxic effect.

It should be noted that we excluded the effect of the “optic filter” which is a result of bioluminescence absorption/reabsorption and can be involved into the bioluminescence suppression. The optical density of fullerenol solutions was <0.1 at the maximum bioluminescence wavelength 490 nm [92], and this effect did not skew the results of the toxicological measurements.

All the bioluminescence measurements were conducted in five replicates for all the solutions. The bioluminescence intensities of the bacteria-based and enzyme-based assays were measured after 1 min pre-incubation.

### 3.3. Luminol Chemiluminescence Assay

We used the luminol chemiluminescence method to evaluate the content of ROS in the experimental bacterial suspensions and enzymatic solutions [93,94]. This technique is used to determine the integral content of ROS, assuming that there occurs the dynamic equilibrium of different ROS forms.

The reagents for the chemiluminescence measurements were the following: luminol (C_8_H_7_N_3_O_2_) and potassium ferricyanide (K_3_[Fe(CN)_6_]) from Sigma-Aldrich (St. Louis, MO, USA), 3% solution of H_2_O_2_ from Tula Pharmaceutical Factory (Tula, Russia), potassium hydroxide (KOH) from Khimreactiv (Nizhny Novgorod, Russia). All the reagents were of chemical grade.

The Stock luminol solution (10^−2^ M) was prepared as follows: luminol powder was dissolved in 5 mL 1N solution of KOH, and then, 5 mL of distilled water was added. The chemiluminescence luminol reaction was initiated by K_3_[Fe(CN)_6_], and the maximum value of the chemiluminescence intensity was determined. The concentrations of luminol and K_3_[Fe(CN)_6_] in the experimental samples were 5.1 × 10^−5^ M and 2.4 × 10^−4^ M, respectively. Chemiluminescence was registered out immediately following the bioluminescence measurements in the same bacterial and enzymatic samples.

All the chemiluminescence measurements were carried out in five replicates.

Preliminarily, the dependences of the chemiluminescence intensity on the concentration of H_2_O_2_ were determined in distilled water and 3% NaCl solution for the enzymatic and bacterial luminescence systems, respectively, and they were used as calibration dependences to evaluate the ROS content in the experimental samples.

The chemiluminescence intensities were measured in the bioluminescence assay systems (bacterial and enzymatic), as well as in the bacteria-free or enzyme-free aqueous solutions. *I^rel^* and *ROS^rel^* were obtained at different concentrations of fullerenols (10^−14^–5 gL^−1^). The optical density of the fullerenol solutions was <0.1 in the maximum of the chemiluminescence light emittance (Abs_425_ < 0.1); hence, the effect of “optic filter” was excluded (See Section 3.2).

The relative values of the ROS content, *ROS^rel^*, were calculated as ratios of the ROS content in the experimental solutions, *ROS_F_*, to that in the control solutions (without fullerenols), *ROS_contr_*:

*ROS^rel^* = *ROS_F_*/*ROS_contr_*, (2)

### 3.4. Equipment

Luminoskan Ascent (Thermo Electron Corporation, Solon, OH, USA) was utilized to measure both the bioluminescence and chemiluminescence intensities. All the luminescence measurements were carried out at 25 °C. The optical density of the fullerenol solutions and that of the bacterial suspensions were measured using a double-beam spectrophotometer UVIKON-943 (KONTRON Instruments, Milano, Italy).

### 3.5. Statistical Processing

GraphPad Prism 8 (GraphPad Software, San Diego, CA, USA) was applied in order to calculate the SD-values for *I^rel^* or *ROS^rel^*. These did not exceed 15% and 20%, respectively.

The statistical dependence between the rankings of two variables was analyzed [95] to reveal correlations between the bioluminescence signal and the ROS concentrations. The correlation coefficients *r* were calculated and the results of the bioluminescence and chemiluminescence assays were statistically processed.

### 3.6. Quantum-Chemical Calculations

Fullerenol models were constructed for two complexes with different oxygen and hydrogen atom contents, C_82_O_20_H_14_ (Gd@C_82_O_20_H_14_, Figure 3a,b) and C_82_O_42_H_32_ (Gd@C_82_O_42_H_32_, Figure 3c). Two valence isomers were constructed for fullerenol C_82_O_20_H_14_ and one isomer for fullerenol C_82_O_42_H_32_. The atomic structures of the obtained models were optimized using the DFTB [83,96,97] with 3ob-3-1 parameters [98]. For the equilibrium geometries, the IR spectra (Figure 4b,d,e) were calculated using the GAMESS program [99].

## 4. Conclusions

The paper reveals differences in the biological effects of two Gd-containing fullerenols which are similar in the size of the carbon carcass (C_82_) but have different numbers of oxygen-containing groups on its surface: 20 and 42 (Gd@C_82_O_20_H_14_ and Gd@C_82_O_42_H_32_, respectively). The study is of practical importance, since Gd-containing fullerenols are perspective agents for MRI and cancer research due to a combination of unique paramagnetic properties of gadolinium with fullerenol features, i.e., solubility in water, low toxicity, and antiradical activity.

The difference in the fullerenol bioeffects was experimentally analyzed using bacterial bioluminescence as a testing physiological parameter. The lower toxicity of Gd@C_82_O_42_H_32_ was found. The bioluminescence activation by Gd@C_82_O_42_H_32_ was observed, while this activation was not found under exposure to Gd@C_82_O_20_H_14._ The effects of fullerenols on luminous bacteria were compared to those on enzymatic system, and the difference was attributed to the fullerenol ability to penetrate through the cellular membrane. The variation in the content of ROS in bacterial suspensions, enzyme solutions, and distilled water at different fullerenol concentrations were monitored and discussed in terms of the antiradical ability of the fullerenols. The additional carboxyl groups on the surface of the carbon cage of Gd@C_82_O_42_H_32_ were determined using the FTIR spectra and confirmed by quantum-chemical calculations. These groups are able to affect the polarity of the environment to a higher degree, in addition to other excessive oxygen substituents. It is supposed to lead to the activation ability and lower toxicity of Gd@C_82_O_42_H_32_, its higher penetrative ability through the cellular membrane, balancing of the ROS content in the bacterial suspensions, and lower aggregation in aqueous media.

To conclude, our study revealed additional points for further investigation of the bioeffects of Gd-containing fullerenols: (i) time-course of the toxic characteristics of fullerenols and conventional MRI contrast agents; (ii) aggregation of fullerenols, resulting in an increase in the ROS content in bacterial suspensions in the concentration interval of 10^−3^–5 × 10^−2^ gL^−1^; (iii) efficiency of ROS suppression by fullerenols in the low-concentration range (<5 × 10^−4^ gL^−1^) in water solutions.

## Data Availability

Data are contained within the article.

## Supplementary Materials

The supporting information can be downloaded at: https://www.mdpi.com/article/10.3390/ijms25020708/s1. References [100,101,102] are cited in the supplementary materials.

## Abbreviations

DFTB3	Density Functional Tight Binding method
*EC*_50_	Effective concentration of fullerenol which inhibited bioluminescence intensity by 50%
FMN	Flavinmononucleotide
FTIR	Fourier-transform infrared spectroscopy
Gd	Gadolinium
I	Bioluminescence intensity
IR	Infrared spectroscopy
MRI	Magnetic resonance imaging
NADH	Nicotinamide adenine dinucleotide, disodium salt, reduced
ROS	Reactive oxygen species
XPS	X-ray photoelectron spectroscopy
