# Supplementary material for: Effects of Endohedral Gd-Containing Fullerenols with a Different Number of Oxygen Substituents on Bacterial Bioluminescence
Source: Int J Mol Sci. 2024 Jan 5;25(2):708. doi: 10.3390/ijms25020708 (PMC10815327; doi:10.3390/ijms25020708)
Supplement: Supplementary file 1 [file ijms-25-00708-s001.zip › ijms-2737146-supplementary.pdf]

CARTESIAN COORDINATES

Atomic Cartesian coordinates for equilibrium structures of studied complexes of C_{82} fullerenols (in Å: X, Y, Z) are presented below.

$C_{82}O_6(OH)_{14}$

Isomer – I_1

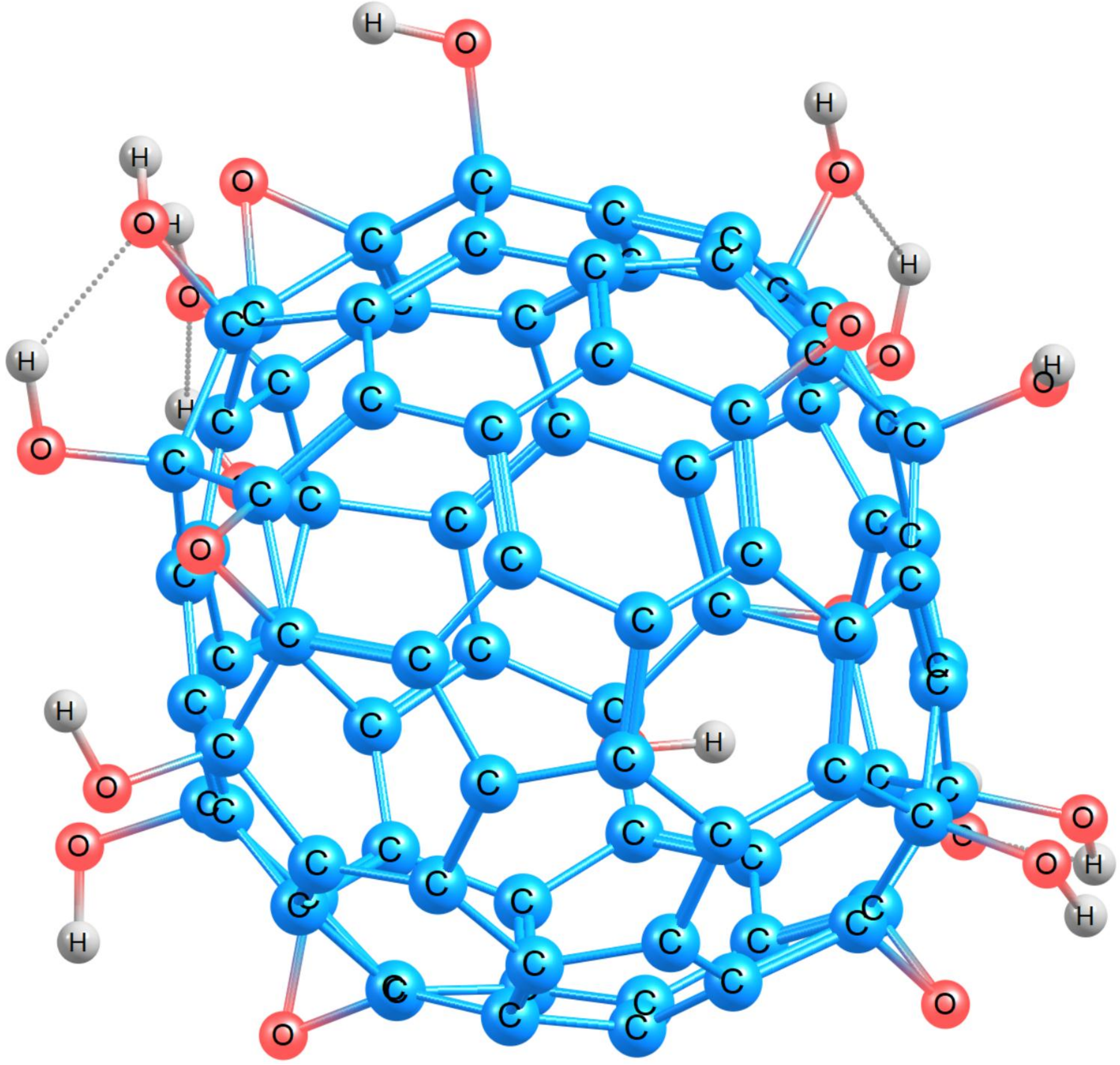

Figure S1. Atomic structure of $C_{82}O_{20}H_{14}$ (Isomer – I_1). Carbon, oxygen, and hydrogen atoms are shown in blue, red, and grey, respectively.

DFTB3/3ob-3-1// Ground state

C	-2.698951913800	-0.000958320600	2.971670907500
C	2.701075522500	-0.135266558400	2.967496399000
C	-3.364705153900	1.257122477900	2.377862926600
C	3.091315848000	0.985409236900	2.154770878500
C	-3.090259171100	-1.138153409200	2.183382211600
C	3.366629163400	-1.406076248700	2.400870441700

C	-3.706068098100	0.724784034800	0.940137722000
C	3.722492905500	0.524290152900	0.976742175700
C	-3.724078832800	-0.703452468400	0.996864617600
C	3.705820798300	-0.905050028900	0.950656014500
C	2.501844403300	3.172709634600	1.060458181800
C	-2.462255308300	3.334888673700	1.055914355900
C	2.461900229000	-3.511601113100	1.124987790300
C	-2.502283098400	-3.348221191700	1.134670277000
C	4.014451509900	1.353522572100	-0.271163678600
C	-3.615611824300	1.434030816500	-0.245938869700
C	3.610454830900	-1.638938731500	-0.219493618000
C	-4.020179862600	-1.560256208300	-0.232152794500
C	3.267654855000	2.729653452400	-0.182640962100
C	-3.389747101400	2.972175332000	-0.168773853100
C	3.386581228700	-3.174898173000	-0.109018810600
C	-3.272548163300	-2.932635045000	-0.118019071500
C	-2.417418220800	2.482974774000	2.330713803700
C	2.373162180200	2.241416038600	2.206251429500
C	-2.371947432900	-2.393585841900	2.259622041500
C	2.419377970200	-2.632380690100	2.381327258200
C	-3.549278882500	0.702377241700	-1.519189443900
C	3.617805778200	0.549824531100	-1.539796961100
C	-3.626997660600	-0.784512881200	-1.520037377100
C	3.541657692100	-0.937075810600	-1.510454739200
C	-2.580009920300	3.605054059600	-1.403573834800
C	2.466983851300	3.234538776800	-1.335409014200
C	-2.473762604500	-3.460009606300	-1.261871360400
C	2.576094170800	-3.831784511600	-1.328731813100
C	2.453401715700	2.521738620400	-2.504045835300
C	-2.500190972700	2.698581884500	-2.623869764600
C	2.502831087100	-2.958741487000	-2.573940483500
C	-2.461215735700	-2.771497003000	-2.445710137100
C	-3.117308478200	1.348273361500	-2.662466477800
C	3.136249547900	1.225795355700	-2.627931201500
C	-3.142411338000	-1.479854750800	-2.594547452200
C	3.104099059500	-1.605437629100	-2.637926658300
C	-2.607747706000	0.666276814600	-3.956802195700
C	2.605215686100	0.603010969700	-3.923848628100
C	-2.624898176400	-0.889267282600	-3.917468715500
C	2.609273650300	-0.955025758100	-3.961913753100
C	-1.228301417800	2.642534637100	-3.482943465700
C	1.262800819200	2.486067585800	-3.338816157500
C	-1.274685334600	-2.756809527400	-3.285333517800
C	1.219067999600	-2.919602672900	-3.439567986400
C	-1.195475986800	1.276022190900	-4.084945428000
C	1.218846595100	1.285059911400	-4.003099972700
C	-1.231237861800	-1.569031462200	-3.972086460600
C	1.187463107900	-1.564624495100	-4.063605917900
C	-1.166240958500	3.881226385000	-0.855978146900
C	1.238551307400	3.859147856100	-0.848289902300
C	-1.243177123300	-4.070303434500	-0.763074663600
C	1.161921370500	-4.092252360800	-0.774065577600
C	-1.173246174300	3.863250544300	0.552153033400
C	1.251090494800	3.854625205600	0.566358064600
C	-1.252997920400	-4.038371268000	0.651758912600
C	1.171586773900	-4.047338980600	0.633587127100

C	1.208382399700	2.291487254900	2.959897557400
C	-1.100767549800	2.386136089400	2.994357564900
C	1.104102928900	-2.521132475700	3.045471841800
C	-1.205591517300	-2.426209122500	3.012034602600
C	-0.676504206500	1.186153033200	3.693726455900
C	0.733094388200	1.133509474600	3.680279476600
C	-0.729470420300	-1.253760846900	3.707511096600
C	0.680389302700	-1.305988319400	3.718296424800
C	1.476010001300	-0.105176694300	3.653752239000
C	-1.472134882500	-0.015992686900	3.655412716300
C	-0.025667168400	0.593064628500	-4.305248794400
C	0.014749589300	-0.888152684100	-4.292232795400
C	0.063986160600	3.421494865100	-3.104136931200
C	-0.073497645000	-3.684211067800	-3.032098556200
C	0.028437244500	3.849101291300	-1.595850332600
C	-0.033945652100	-4.073174284700	-1.511869274600
C	0.038501803000	3.778949865500	1.292342896300
C	-0.039035584700	-3.948146227100	1.373572560100
C	0.031391661300	3.075525233100	2.556080928100
C	-0.029013000600	-3.217556037200	2.621721330300
O	-3.222092567200	4.835796995200	-1.767105371400
H	-3.205522050900	4.918727373800	-2.738289745400
O	-4.661190355000	3.579968920300	-0.048777625200
H	-4.593008787300	4.495089192800	-0.380219336100
O	-5.432171384000	-1.840339608700	-0.309192889900
H	-5.632393872200	-2.574071685700	0.299129604800
O	-3.796002174400	-3.903063961900	0.794324974100
O	4.659219279300	-3.778594023900	0.021381903900
H	4.600064390600	-4.685359487900	-0.336691401500
O	3.215549574600	-5.072219671900	-1.663809036600
O	5.425764575900	1.632396537800	-0.364901446200
H	5.639342480300	2.343117136500	0.265757999700
O	2.376147747800	-3.696664593100	-3.799885394200
O	-0.197131235500	-4.854666729200	-3.848339246800
O	-3.407473831900	-1.403480270900	-4.994044359400
H	-3.811631048600	-0.655252973700	-5.472754667100
O	-3.406035845600	1.104045822000	-5.071915895300
H	-3.376941883900	2.080922660800	-5.091084303000
O	4.550204591000	-1.740256577100	3.118311770000
O	3.062384655800	-3.922560058200	2.350010793600
O	3.399834442700	-1.431184230200	-5.043580241800
H	3.783745630600	-0.668980018700	-5.514974493100
O	3.402308914400	1.071686222900	-5.032307099000
H	3.460348340200	2.041827177800	-4.995674873400
O	0.193572056300	4.564742171400	-3.958235913200
H	-0.686244374800	4.957211611000	-4.091188890100
O	-2.398771284900	3.409216123600	-3.886234389200
O	-3.060426631100	3.772672585300	2.272494875900
O	3.793978856200	3.721224914200	0.706319993300
H	3.214117574000	-5.166936923400	-2.634931666000
O	-4.547489329100	1.606961437500	3.089221509200
H	-4.772130812700	2.528732326200	2.864998367500
H	0.692269435200	-5.177079031800	-4.074095180000
H	4.769176910400	-2.669486842300	2.919794741100

$C_{82}O_5(OOH)_1(OH)_{13}$

Isomer – I_2

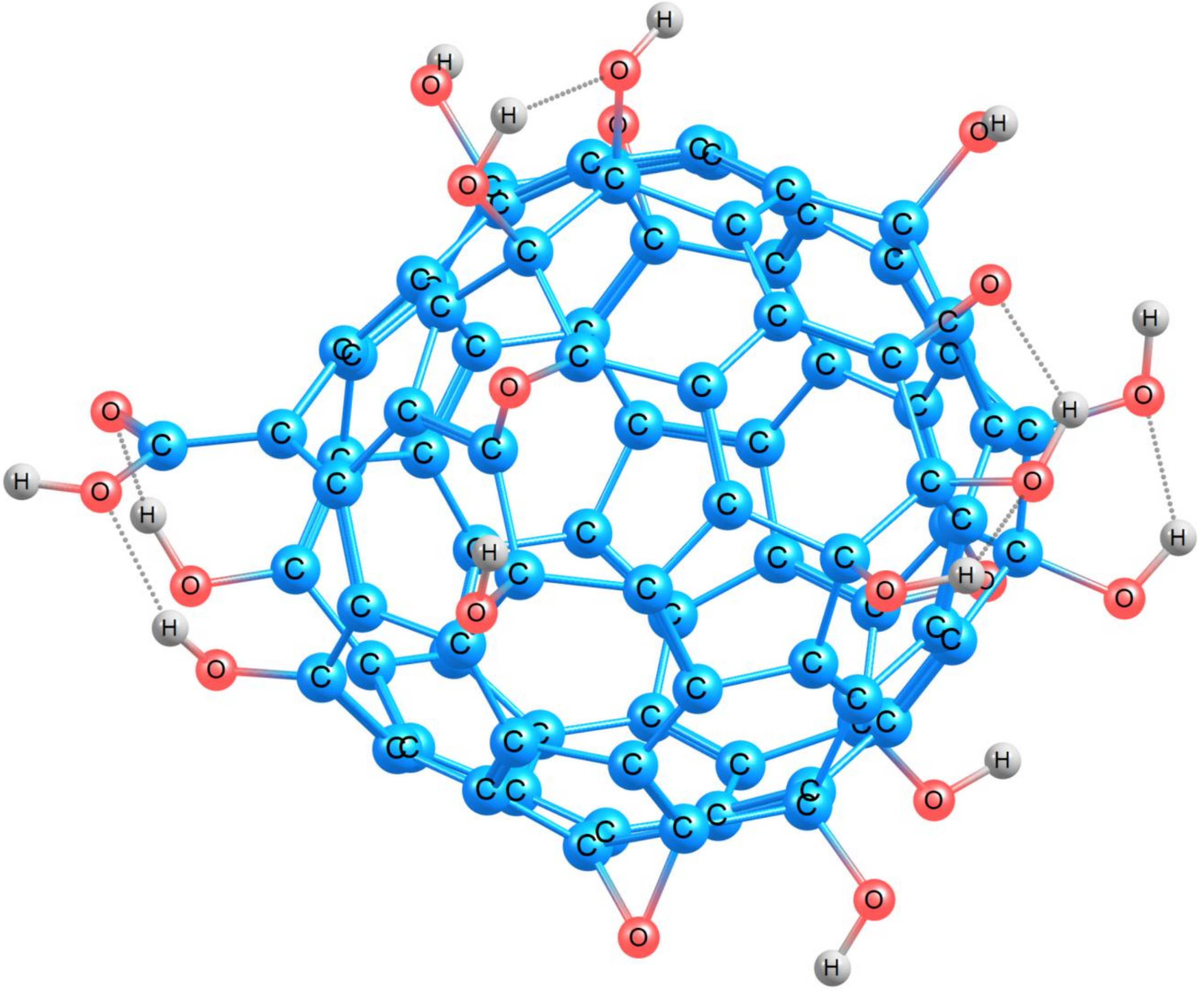

Figure S2. Atomic structure of $C_{82}O_{20}H_{14}$ (Isomer – I_2 ($C_{82}O_5(OOH)_1(OH)_{13}$)). Carbon, oxygen, and hydrogen atoms are shown in blue, red, and grey, respectively.

DFTB3/3ob-3-1// Ground state

C	-2.729787734500	-0.041683268800	2.979925608500
C	2.604649220200	-0.166588485200	2.871308331600
C	-3.393897003000	1.216893029200	2.389404321200
C	3.063855803500	0.966055041700	2.142179725400
C	-3.117470233800	-1.161964719000	2.223005471100
C	3.113985351800	-1.356394926700	2.276837702900
C	-3.779890047000	0.679551304400	0.965141852500
C	3.779138675400	0.485695693300	1.003121629100
C	-3.799465807100	-0.699551664400	1.005781768000
C	3.805919639200	-0.935579842100	1.030635092200
C	2.486271475400	3.149414077700	1.092752528400
C	-2.487235942700	3.283991569100	1.058002263500
C	4.385710958400	-3.987428897800	0.393601405300
C	-2.514294959900	-3.312675245400	1.131022815400
C	4.054659710200	1.341793977500	-0.239978559700
C	-3.713319809700	1.423224240300	-0.264859343800

C	3.740048508600	-1.663154784200	-0.233826185200
C	-4.068282297400	-1.551456595400	-0.236591619000
C	3.267074290200	2.703146570000	-0.147974301500
C	-3.420150082700	2.953152019300	-0.176053975700
C	3.459492363600	-3.086622615500	-0.271669302800
C	-3.290147185400	-2.904482729300	-0.123378744400
C	-2.445920982300	2.439003750900	2.332596496300
C	2.351505240100	2.209052839600	2.224691849000
C	-2.378065948300	-2.385681532500	2.262063837900
C	2.550494748200	-2.592120653700	2.760148729300
C	-3.654821672200	0.723221543200	-1.480546300500
C	3.658356735400	0.550236152800	-1.511998970500
C	-3.678307250300	-0.751049485000	-1.515893152300
C	3.590279573400	-0.917341679600	-1.461495785200
C	-2.572859787500	3.551617911900	-1.400125949000
C	2.460605738000	3.199712853000	-1.301038386800
C	-2.468095606500	-3.412190210000	-1.253688606100
C	2.424858458900	-3.548083219700	-1.208384954800
C	2.439793245600	2.484062788400	-2.472372943900
C	-2.433302833100	2.607463353500	-2.568169778400
C	2.354511883300	-2.823792773000	-2.402656094900
C	-2.445111674100	-2.700965127100	-2.427535520200
C	-3.021806579300	1.378369338800	-2.621848587100
C	3.153026320100	1.214468703100	-2.604421137300
C	-3.149964991800	-1.430887019700	-2.588983270500
C	3.033349793000	-1.592220216500	-2.562977268700
C	-2.624171757600	0.695988436900	-3.953872871400
C	2.634074541800	0.585476118600	-3.902284622600
C	-2.630249775500	-0.851430364600	-3.921116951000
C	2.660582219700	-0.966622207100	-3.918176771800
C	-1.237087667100	2.666027170600	-3.492082588300
C	1.261502803300	2.448400487300	-3.321303788800
C	-1.262904008100	-2.685632235200	-3.257707865900
C	1.215475841200	-2.901325082200	-3.369841276100
C	-1.232916347100	1.357237766300	-4.247630592500
C	1.237210540700	1.249630373100	-4.017138283700
C	-1.229417274400	-1.513556702700	-3.994631933700
C	1.249377768000	-1.627379712000	-4.193284188100
C	-1.174584241300	3.841834998200	-0.839933343300
C	1.229644303500	3.819309618600	-0.818488814500
C	-1.258286196900	-4.057679601200	-0.749845646800
C	1.178431787100	-4.237804835300	-0.745163792600
C	-1.191782261700	3.814124596700	0.567344239400
C	1.233463187700	3.820061210200	0.595497301100
C	-1.275533913200	-4.002864234900	0.671961829600
C	1.056146517000	-4.527214163000	0.663044435900
C	1.183059887800	2.260774870300	2.991970845300
C	-1.136398411400	2.345462322800	3.017186668500
C	1.135806953700	-2.560765444000	3.164003438400
C	-1.188477842800	-2.444969636600	3.006392404600
C	-0.720221245500	1.154416653200	3.721169378200
C	0.704921479300	1.109513022300	3.713700185000
C	-0.759261208000	-1.284522162500	3.719432841700
C	0.664354034000	-1.321876063200	3.753028692100
C	1.421237768600	-0.125346540100	3.680926285200
C	-1.493189455900	-0.054008972700	3.685416108800

C	0.027641666900	0.563868335300	-4.404073279000
C	-0.010956848500	-0.841105721800	-4.379872832700
C	0.066023476500	3.397532680500	-3.064047395600
C	-0.078966610100	-3.617867787300	-2.952138753900
C	0.025940272500	3.804323983300	-1.571151370800
C	-0.040603495400	-4.029551730800	-1.463660558000
C	0.015929209800	3.734541954900	1.314455391400
C	-0.084714990300	-4.065044943100	1.422151549000
C	0.005207318700	3.027395396800	2.575448093000
C	-0.001404449100	-3.255579408000	2.648537741300
O	-3.212483344800	4.781746362900	-1.804400839300
H	-2.655475542700	5.227921616400	-2.464606881000
O	-4.667901524000	3.611700514900	-0.055465659000
H	-4.596993518000	4.487546254800	-0.477712508200
O	-5.470540352800	-1.866005624900	-0.339140901500
H	-5.672805840200	-2.574930464000	0.296538700800
O	-3.796901699200	-3.888455099400	0.782770263900
O	5.229355860400	-3.682486929100	1.261751275900
H	4.186172270200	-3.572717191400	2.623831036800
O	2.047785403300	-5.117489373900	1.309206697800
O	5.455040929600	1.665856578000	-0.354321362300
H	5.652908249200	2.374351687000	0.283574829000
O	1.442089871900	-2.927488545200	-4.785817400100
O	-0.196142202700	-4.824231483400	-3.734143882200
O	-3.401969294100	-1.368274692400	-5.004682223900
H	-3.944537774100	-0.640986382100	-5.366586030500
O	-3.553312782800	1.132389530000	-4.953728147300
H	-3.245580465500	2.002853143300	-5.280123184700
O	4.383861965400	-5.284155130200	-0.070635095600
O	3.247156463900	-3.678300681200	2.923550944600
O	3.594003382400	-1.441808742400	-4.877865780600
H	3.825804425900	-0.712947777000	-5.481579525500
O	3.431270998100	1.052594022800	-5.014255566400
H	3.547858960200	2.014599635500	-4.936520170700
O	0.197546457800	4.582259453800	-3.870281871400
H	-0.011826326400	4.342704603100	-4.791186695600
O	-1.430208966100	2.624794549700	-4.930281119500
O	-3.091337738200	3.726113108100	2.275484227700
O	3.773682669100	3.706287534000	0.742801957800
H	2.706908016300	-5.499248524400	0.696704525700
O	-4.563631634100	1.577698165100	3.120692645400
H	-4.777389242800	2.503392802600	2.899093781200
H	0.114720145100	-4.623184301900	-4.636492140500
H	5.157049342000	-5.745714571900	0.300754352100

$C_{82}O_8(OOH)_2(OH)_{30}$

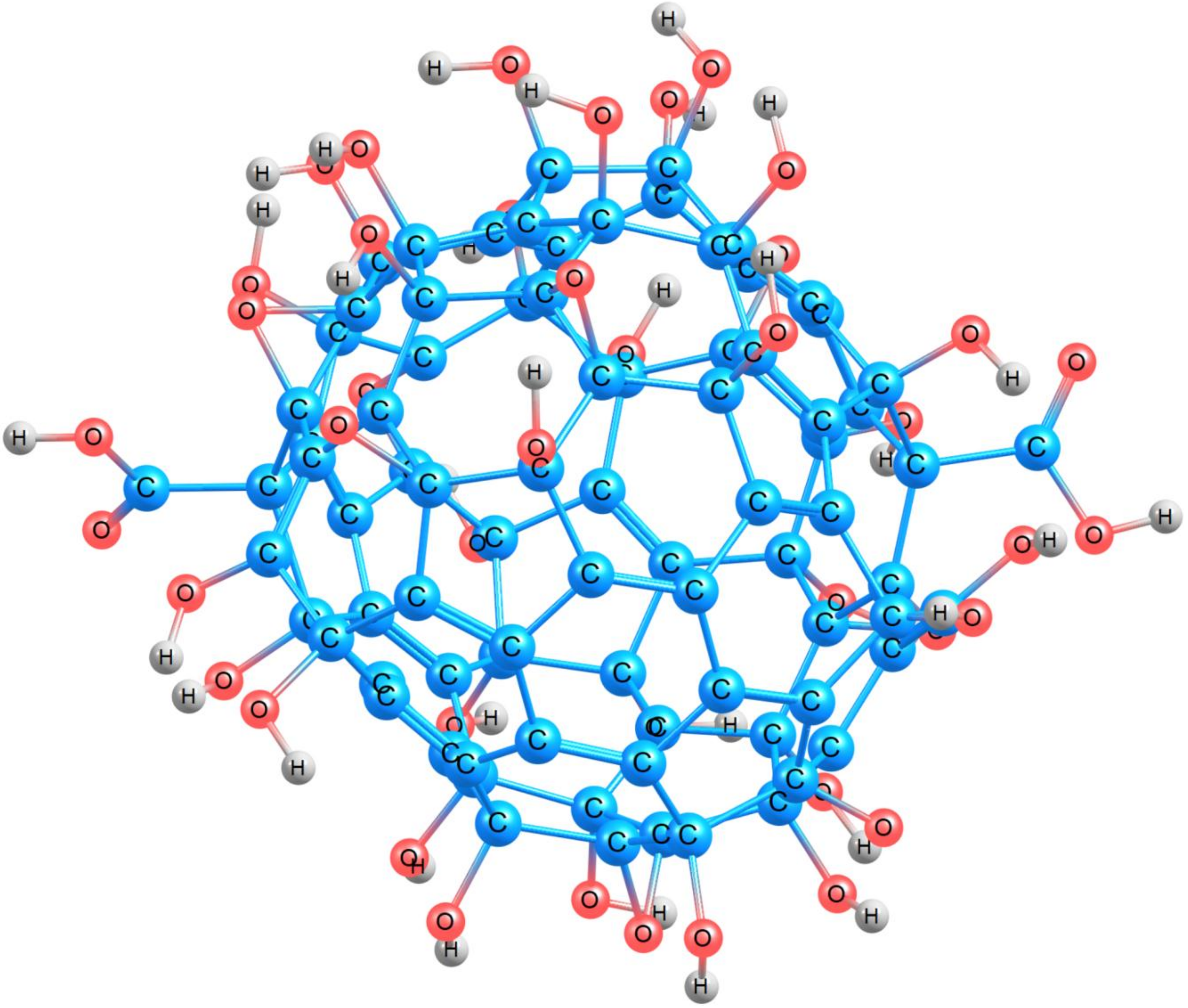

Figure S3. Atomic structure of $C_{82}O_{42}H_{32}$. Carbon, oxygen, and hydrogen atoms are shown in blue, red, and grey, respectively.

DFTB3/3ob-3-1// Ground state

C	-2.631507149796	0.088513923982	3.138417932570
C	2.644213942815	-0.135892783955	3.124230423607
C	-2.861853454726	1.219169912423	2.378691770906
C	3.268019888505	1.060932571503	2.427489139920
C	-3.258732073548	-1.102546749525	2.434859332892
C	2.870769655700	-1.260299756417	2.354187987934
C	-3.272019928535	0.813847877626	0.995651462525
C	3.433430666464	0.517582102755	0.997943779573
C	-3.430734685443	-0.547552649778	1.010677937565
C	3.274617109517	-0.843650591639	0.972576144535
C	2.417190360881	3.251805111502	1.298286724386
C	-2.525774117860	3.547972696371	1.824458491198
C	2.531836650878	-3.584332780380	1.782577769189
C	-2.413705175918	-3.284225095495	1.283813540409
C	3.874593617250	1.433879881357	-0.134988901958
C	-3.673799375337	1.577409823284	-0.334867180864
C	3.669485113349	-1.596194081274	-0.366041247821
C	-3.878078003262	-1.454548034325	-0.127202987921
C	3.191534608536	2.891751193679	-0.009788692018

C	-3.990075710167	4.124048128142	-1.255630783426
C	3.980375909185	-4.135535299103	-1.309159656401
C	-3.194738856526	-2.913548684664	-0.017387720018
C	-2.480497381859	2.596375505842	2.992409478663
C	2.439122322896	2.379474801943	2.514636568857
C	-2.429602213892	-2.421840171887	2.507331745839
C	2.492420203863	-2.642431053803	2.958567950639
C	-3.537254181399	0.582936032715	-1.515652057288
C	3.517753689411	0.763806377657	-1.482432537326
C	-3.528301609385	-0.773555649673	-1.470952166346
C	3.526409707426	-0.592256696719	-1.538161121278
C	-2.857000814684	3.036287047634	-1.048281056498
C	2.155129802000	3.150019835562	-1.055461969542
C	-2.163823993009	-3.163687142545	-1.070422982499
C	2.848659497692	-3.049844681627	-1.087388479504
C	2.388443363894	2.872298747705	-2.503099639889
C	-2.530292196857	2.489031751866	-2.419242793892
C	2.514870151875	-2.491081695892	-2.451976344880
C	-2.404296744904	-2.873875808674	-2.514507268841
C	-3.082051383587	1.287337260430	-2.686618088804
C	3.323193910469	1.623729749257	-2.767302319757
C	-3.340366714461	-1.623052865250	-2.763733348731
C	3.065213615629	-1.287216423440	-2.712382038760
C	-2.520718477858	0.631751033703	-3.888241559257
C	2.552669195827	0.896627991593	-3.988461037183
C	-2.575893813822	-0.886084290590	-3.982900163192
C	2.497915564864	-0.621915568714	-3.905810871210
C	-1.507835084304	2.892563364708	-3.486356937417
C	1.023057634538	2.512623631837	-3.114263258572
C	-1.041950490547	-2.509127662876	-3.129501352599
C	1.487082683301	-2.885978885701	-3.517290340383
C	-1.353222668395	1.479649234333	-4.305810722071
C	1.109518143515	1.446178949338	-3.927822837205
C	-1.132432832502	-1.436047679347	-3.933817820218
C	1.328333455417	-1.466418867311	-4.324378929056
C	-1.562772549274	3.741344870278	-0.471909260808
C	1.115497112513	4.166864715091	-0.646963337695
C	-1.121836714483	-4.183481491116	-0.675342313707
C	1.557277969285	-3.759397569296	-0.510244579758
C	-1.426585171339	3.751935615296	1.026971133555
C	1.212115434430	4.221059568111	0.946817535565
C	-1.210514144445	-4.250579608073	0.918408046558
C	1.428624294377	-3.782202503271	0.989139320567
C	1.354653666390	2.524165147873	3.639296175353
C	-1.096528983526	2.409621271931	3.577307984362
C	1.111339621507	-2.460464924863	3.551723684367
C	-1.339464211371	-2.575645720817	3.625277967337
C	-0.646470038716	1.202626082477	4.072074566158
C	0.801637135625	1.165425885451	4.078741910141
C	-0.784221017627	-1.220513452447	4.073052796130
C	0.663827178692	-1.257609299424	4.058713882147
C	1.476083485309	-0.041613509988	3.988109686192
C	-1.459066760347	-0.012777125015	3.995605953209
C	-0.009488206019	0.864253457629	-4.789989162824
C	-0.017734849974	-0.847193087609	-4.796854534830
C	-0.152329887952	3.447001195448	-2.846341512689

C	0.134770262958	-3.445581934446	-2.875023127711
C	-0.294642910859	3.843469975275	-1.306981671431
C	0.284871790859	-3.854506219273	-1.339698483378
C	-0.089615926978	3.852451703262	1.722551008227
C	0.095127895942	-3.888406251229	1.690629397229
C	0.045045818978	3.225601466530	3.117281082603
C	-0.032529487964	-3.272857641520	3.091036926572
O	-5.021332816741	1.888716596147	-0.199392900916
O	-3.706892168302	5.348965361587	-0.763038672651
O	4.266842261064	3.842095864258	-0.193596121930
O	5.287035858576	1.585777862299	-0.038869726957
O	5.017599499719	-1.908545355145	-0.240602217913
O	3.699782221314	-5.364449580557	-0.824937046652
O	-5.290081239618	-1.607106005291	-0.024645662007
O	-4.271342809055	-3.862032431267	-0.203211405897
O	-3.214999207567	-1.276952193431	-5.191893058622
O	-2.508919304838	1.336106344391	-5.147352529673
O	3.506088260384	-3.026852866643	3.903887145219
O	-2.043292750079	3.869080426257	-4.341377177053
O	0.175138756942	4.684117011883	-3.502196108430
O	2.479769234863	-1.315999407390	-5.170535193649
O	3.185716373529	1.297199048389	-5.197458512642
O	-0.196164180913	-4.677346277884	-3.539231120396
O	2.018226806082	-3.855452100269	-4.382936998992
O	-3.222520327553	-3.620101458384	2.413715251929
O	-4.560633276934	-1.346891088382	2.979546691652
O	4.572216002912	1.301215478414	2.968125588630
O	3.231746324552	3.578417506384	2.426833240913
H	1.285576186433	-4.280014036047	-4.864571089808
H	-1.313158482393	4.296446900064	-4.824331459798
H	5.587992754463	2.095269050026	-0.813343572607
H	3.880143566218	4.722425717874	-0.343979384857
H	-3.885863441251	-4.741321066838	-0.362428886854
H	-5.595302861475	-2.109944659066	-0.801815839628
O	5.023801989747	-3.910587126209	-1.895461359133
O	3.796489509305	-3.839051288253	1.394732115359
O	-5.036454337730	3.903780263216	-1.838442465149
O	-3.792391196297	3.806549266282	1.445426922320
H	-5.187580933663	2.410547954905	0.599563252740
H	5.189479469653	-2.432121104900	0.556067146746
H	-4.426169112004	5.958726275320	-0.995991964536
H	4.417942284997	-5.972118643289	-1.066602628520
H	-3.085353559584	-0.569102757754	-5.848651145350
H	3.052611835612	0.594683785740	-5.859236964364
H	5.048565868734	1.896831217152	2.360717587926
H	-5.042341316725	-1.932342351097	2.366584869938
O	-3.489458994401	2.972733206640	3.945860203207
H	-4.356120921050	3.687090326328	2.234648213986
H	-3.527596689408	2.313767461963	4.659963097889
H	4.364328104045	-3.725743077331	2.182019339005
H	3.547252836397	-2.374290171917	4.623776731922
O	1.192140532445	-4.962184563727	-1.223161555469
O	-1.201386738459	4.950034452738	-1.176800789448
H	-0.265312639891	5.405692969530	-3.013380846650
H	0.245358788894	-5.403126999528	-3.057649829616
O	-0.262706945875	-1.218781082453	-6.144776218235

O	0.228812948921	1.246708672442	-6.136033196231
H	-0.611651869734	1.185124184477	-6.622183773009
H	0.575551908728	-1.154194411455	-6.634341007993
O	4.612296212931	2.037394114051	-3.165243051559
O	2.945281661647	4.014739898193	-3.185077530540
O	-4.631432227898	-2.033439752072	-3.158492897581
O	-2.964639075643	-4.010525890158	-3.203263769554
H	-4.528155515941	-2.627862800804	-3.923577474201
H	-2.296733572979	-4.725289652874	-3.185663385530
H	4.505137828952	2.638098444817	-3.924869314243
H	2.277639193943	4.729418968843	-3.157654990546
O	-1.915785201125	-3.282129667521	4.711851722850
O	0.143000008961	-4.673577779888	2.931525772668
O	1.936495245140	3.221772764515	4.728645857868
O	-0.131326719958	4.627548507906	2.970015189673
H	2.601641904812	3.841117538241	4.375435025010
H	-2.583100914843	-3.898215205232	4.357023430043
O	-1.501985391297	-5.471128233504	-1.215013178459
O	-1.568908948291	-5.580085949461	1.279149547400
O	1.493527361299	5.458785718535	-1.177908748492
O	1.572023489307	5.547680326492	1.316487272407
H	1.467101251322	6.095981943214	-0.433184535826
H	1.197540255454	5.727115175380	2.203493137982
H	-1.189432802472	-5.767004163390	2.162495852043
H	-1.476197564335	-6.113474889229	-0.474600692810

CHARACTERIZATION OF Gd-CONTAINING FULLERENOLS

The assessment of water in the samples was performed by thermogravimetric analysis (TGA). The weight loss of Gd@$C_{82}O_x(OH)_y$, x+y=40-44 and Gd@$C_{82}O_x(OH)_y$, x+y=20-24 was observed from room temperature to 900 ^{0}C at a rate of 10 ^{0}C min^{-1} under Ar flow at 90 mL min^{-1} (Figure S4 a,b).

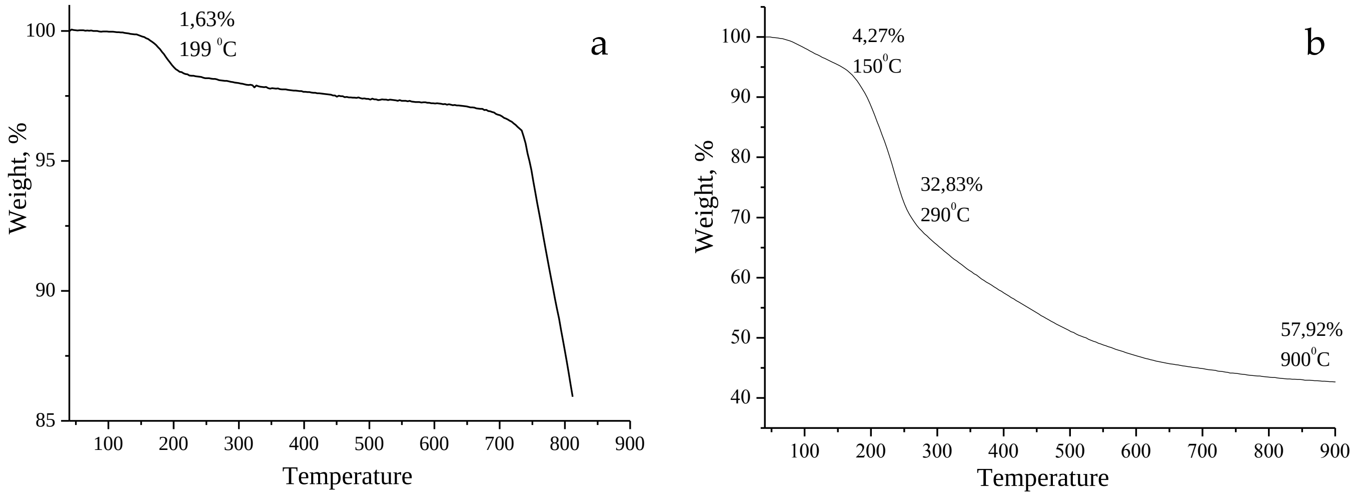

Figure S4. TGA chart for measuring the weight loss (wt%) of Gd@$C_{82}O_x(OH)_y$, x+y=40-44 (a) and Gd@$C_{82}O_x(OH)_y$, x+y=20-24 (b)

In Figure S4a, an initial weight loss (1,63 wt%) of the sample Gd@$C_{82}O_x(OH)_y$, x+y=40-44 is evident from 140-200ºC; it indicates the loss of bound water molecules. The weight degradation observed higher than 731ºC, it is attributed to the sublimation of C_{82} molecules [100-101]. An initial weight loss of the sample Gd@$C_{82}O_x(OH)_y$, x+y=20-24 was observed from 120-180ºC (Figure S4b), indicating the loss of bound water molecules (4.27%). Weight

loss (32.33 wt%) at 210-290ºC corresponds to CO_2 loss. Next, a gradual weight loss occurs (57.92 wt%), which is apparently due to the sublimation of C_{82} molecules.

The study of fullerenols (sample 1(S1) and sample 2(S2)) were carried out by X-ray photoelectronic spectroscopy (XPS) using UNI-SPECS spectrometer, SPECS Gmbh. Figure S5 shows the carbon C1s spectrum lines; three lines were found in both samples after Lorentz decomposition. The line with the maximum at 284.7 eV corresponds to the bond between the carbon atoms in the C_{82} molecule. The line with the maximum at 286.2 eV corresponds to the hydroxyl bond C-OH, and the peak is at 289 eV does to carbonyl bond C=O. The peaks areas of C 1s from XPS spectra of fullerenols (S1 and S2) are presented in Table S1.

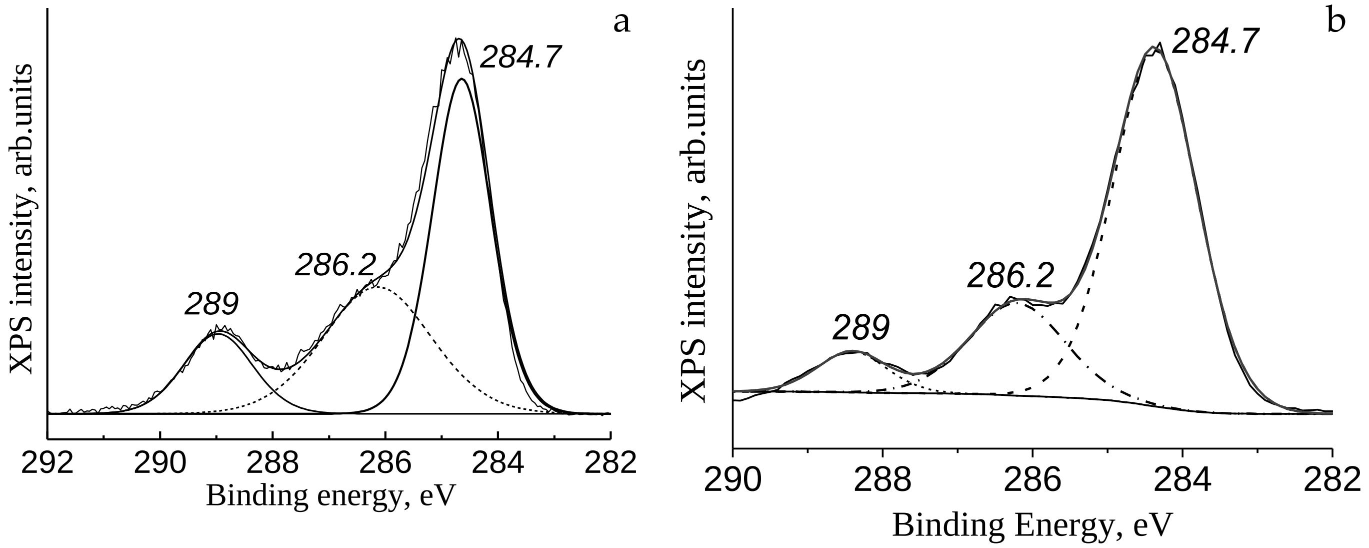

Figure S5. XPS C1s line of S1 (a) and S2 (b)

Table S1. The peaks area analysis of C1s from XPS spectra of fullerenols

Sample	Peak area (%)		
	C-C	C-O	C=O
S1	50.6	35.2	14.2
S2	72.6	19.3	8.1

The number of these functional groups was calculated on the basis of XPS of S1 and S2, based on the proportion of carbon atoms chemically bonded with oxygen. Given that the number of -OH groups attached to the fullerene must be even [102], the composition of the product, determined on the basis of the XPS analysis, can be presented as S1 – $Gd@C_{82}O_x(OH)_y$, where x+y=40-42 and S2 – $Gd@C_{82}O_x(OH)_y$, where x+y=20–24.
